# The value of family history in the diagnosis of hypersensitivity pneumonitis in children*

**DOI:** 10.1590/S1806-37132014000200013

**Published:** 2014

**Authors:** Joana Cardoso, Isabel Carvalho

**Affiliations:** Vila Nova de Gaia/Espinho Hospital Center, Porto, Portugal; Vila Nova de Gaia/Espinho Hospital Center, Porto, Portugal

**Keywords:** Alveolitis, extrinsic allergic, Bronchoalveolar lavage, Glucocorticoids

## Abstract

Hypersensitivity pneumonitis (HP), or extrinsic allergic alveolitis, is an immunologically mediated disease resulting from the inhalation of organic substances that trigger an inflammatory response in the alveolar wall, bronchioles, and interstitium in susceptible individuals. Although HP is predominantly an occupational disease, seen in adulthood, cases in children have been described. The diagnosis of HP requires a high degree of suspicion. The treatment consists in avoiding contact with the antigen, and, in some cases, systemic corticosteroids might be necessary in order to prevent its progression to pulmonary fibrosis. We report the clinical cases of three children with a history of contact with birds and a family history of HP. All three patients presented with cough and dyspnea on exertion. The disease was diagnosed on the basis of the clinical history and ancillary diagnostic test results consistent with the diagnosis, including a predominance of lymphocytes (> 60%, CD8+ T lymphocytes in particular) in bronchoalveolar lavage fluid and a ground-glass pattern seen on HRCT of the chest. Early diagnosis is crucial in order to prevent HP from progressing to pulmonary fibrosis. Hereditary factors seem to influence the onset of the disease.

## Introduction

Hypersensitivity pneumonitis (HP), or extrinsic allergic alveolitis, is an immunologically mediated disease caused by the inhalation of organic substances that, in susceptible individuals, trigger an inflammatory response in the alveolar wall, bronchioles, and interstitium.^(^ ^1^ ^-^ ^8^ ^)^ Although HP is predominantly an occupational disease, seen in adulthood, cases in children have been described, most of which occurred after exposure to avian proteins.^(^ ^4^ ^,^ ^6^ ^)^

The prevalence of HP ranges from 5-15% in individuals exposed to one allergen,^(^ ^1^ ^-^ ^3^ ^)^ and the frequency of the disease is related to several factors (the amount of allergen inhaled, the duration of exposure, the nature of the antigen, and the host immune response).^(^ ^1^ ^,^ ^2^ ^)^ Heredity may play an important role, with families positive for HLA-DR7, HLA-B8, and HLA-DQw3 showing a stronger predisposition.^(^ ^2^ ^,^ ^6^ ^)^

HP can be classified on the basis of symptoms (as acute, subacute, or chronic) or on the basis of the dynamic nature of the disease (as acute progressive, acute intermittent non-progressive, or recurrent non-acute disease).^(^ ^1^ ^-^ ^3^ ^,^ ^5^ ^-^ ^6^ ^,^ ^8^ ^)^ The acute form corresponds to intermittent, intense allergen exposure, and it is similar to a respiratory infection of viral etiology that resolves within 24-48 h.^(^ ^1^ ^-^ ^3^ ^,^ ^5^ ^-^ ^7^ ^)^ The subacute form is characterized by dyspnea on exertion, asthenia, and weight loss, and it corresponds to continued but less intense allergen exposure.^(^ ^1^ ^-^ ^3^ ^,^ ^5^ ^-^ ^6^ ^)^ The chronic form is characterized by progression to irreversible lung injury, with pulmonary fibrosis,^(^ ^1^ ^,^ ^3^ ^)^ and it corresponds to prolonged insidious inhalation of low concentrations of antigen, with no history of acute disease.^(^ ^1^ ^-^ ^3^ ^,^ ^5^ ^-^ ^7^ ^)^

There is no pathognomonic diagnostic test, and a presumptive diagnosis is made on the basis of a high index of suspicion, clinical history, physical examination, laboratory test results, pulmonary function testing (PFT), and imaging study results.^(^ ^1^ ^-^ ^4^ ^,^ ^6^ ^)^

Laboratory data are nonspecific-leukocytosis, increased ESR, and increased levels of C-reactive protein (CRP) and immunoglobulin.^(^ ^1^ ^,^ ^2^ ^,^ ^4^ ^)^ Skin tests and the presence of precipitating antibodies to the antigen are markers of exposure, but negative results do not exclude the diagnosis.^(^ ^1^ ^-^ ^4^ ^,^ ^6^ ^)^ Inhalation challenge testing with the predisposing antigen is the best diagnostic method when diagnostic questions persist, but it must be performed in a hospital setting.^(^ ^1^ ^-^ ^4^ ^,^ ^6^ ^)^

Usually, PFT shows a restrictive pattern, characterized by a decrease in FVC and TLC. In the chronic phase, it is possible to find an obstructive pattern, and DLCO is usually decreased.^(^ ^1^ ^-^ ^4^ ^,^ ^6^ ^)^

Bronchoalveolar lavage (BAL) fluid shows a predominance of lymphocytes (> 60%), namely suppressor T lymphocytes (CD8+), with a decrease in the CD4/CD8 index < 1% (rarely found in children).^(^ ^1^ ^-^ ^4^ ^,^ ^6^ ^)^

Chest X-ray changes appear as a function of the degree of disease and are little related to symptom severity, ranging from normal to a nodular/reticulonodular pattern (in acute and subacute forms).^(^ ^1^ ^-^ ^6^ ^)^ Chest HRCT can show a diffuse micronodular pattern (acute phase) or obstructive emphysema, interstitial fibrotic lesions, and ground-glass areas (chronic phase),^(^ ^1^ ^-^ ^7^ ^)^ being the most useful test for diagnosis.

The treatment consists in avoiding contact with the antigen, which can be the sole treatment in acute forms. Systemic corticosteroids are the treatment of choice in subacute and chronic forms.^(^ ^1^ ^-^ ^4^ ^,^ ^6^ ^)^ The prognosis varies from full recovery in acute and non-progressive forms to pulmonary fibrosis in chronic forms. The degree of pulmonary fibrosis at diagnosis is the major prognostic factor.^(^ ^1^ ^,^ ^2^ ^)^

## Case reports

Case 1

**Figure 1:**
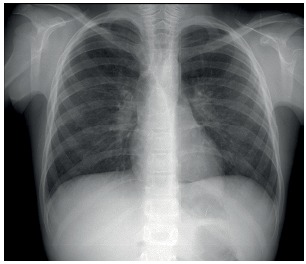
Chest X-ray showing bilateral perihilar and basilar reticulonodular infiltrate.

We report the case of a 12-year-old boy with a history of asthma and a family history of HP. Living in a rural area, the boy had contact with pigeons and canaries, which aggravated the symptoms. He presented to the emergency room with a 2-day history of productive cough and fever, accompanied by anorexia and weight loss. A chest X-ray showed a bilateral perihilar infiltrate, and the patient was discharged after being treated with clarithromycin, which was replaced by a combination of amoxicillin and clavulanic acid 5 days later because of persistence of symptoms. One month later, because the patient continued to have an intermittent cough with periods of worsening and dyspnea on minimal exertion, he again sought medical attention, presenting with perioral cyanosis, pallor, hypoxemia (SpO_2_ = 85% on room air), overall retraction, and an overall decrease in breath sounds, with crackles at the lung bases. Ancillary test results were as follows: ESR, 36 mm/h; PCR, 22.8 mg/L; IgA and IgG levels, increased; Phadiatop^(r)^ test (Phadia, Uppsala, Sweden) for inhalation and food allergy, negative; PPD, 0 mm induration; sweat test, 34 mEq/L; chest X-ray findings, bilateral perihilar and basilar reticulonodular infiltrate (Figure 1); echocardiogram, unremarkable; chest HRCT findings, air-space consolidation and air bronchogram in both lower lobes, accompanied by a diffusely distributed bilateral interstitial pattern, a centrilobular pattern with some micronodules, and ground-glass areas; serology for *Chlamydophila psittaci*, negative; PFT, indicative of mild obstructive lung disease; DLCO, moderate defect; BAL fluid findings, an intense lymphocytic alveolitis, with a marked predominance of CD8+ (CD4/CD8 ratio < 0.3%). The patient received oral corticosteroids for a year, which resulted in complete resolution of the symptoms and complete resolution of the lesions seen on HRCT of the chest, as well as in improvement in PFT results by the end of a three-year follow-up period.

## Case 2

**Figure 2:**
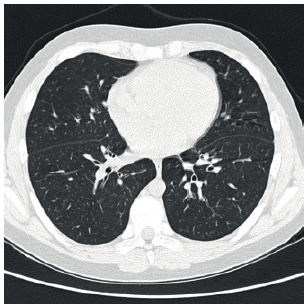
HRCT scan of the chest showing mild, slightly heterogeneously distributed parenchymal thickening in both lungs; small nodules with ill-defined borders; and ground-glass changes.

A 14-year-old girl who lived in a rural area and had birds at home presented with a family history of HP. The patient had been healthy until November of 2002, when she sought emergency room treatment due to fever and cough. Physical examination revealed crackles and respiratory distress. A chest X-ray showed an interstitial infiltrate. The patient was discharged on clarithromycin and prednisolone. She returned to the outpatient clinic one week later, reporting dyspnea on minimal exertion. Ancillary test results were as follows: ESR, 24 mm/h; IgG level, increased; levels of other immunoglobulins, no abnormalities; serologies for *Mycoplasma pneumoniae* and *Chlamydia pneumoniae*, negative; IgG and IgM, negative; chest X-ray findings, increased bronchovascular markings bilaterally and bilateral, perihilar predominant diffuse interstitial infiltrate; chest HRCT findings, mild, slightly heterogeneously distributed parenchymal thickening in both lungs, small nodules with ill-defined borders, and ground-glass changes (Figure 2); echocardiogram and electrocardiogram, unremarkable; BAL fluid findings, an intense lymphocytic alveolitis and a mild neutrophilic alveolitis, with a marked predominance of CD8+ (CD4/CD8 ratio < 0.1%). The patient was maintained on inhaled corticosteroids for 2 years, which resulted in progressive clinical improvement.

## Case 3

**Figure 3:**
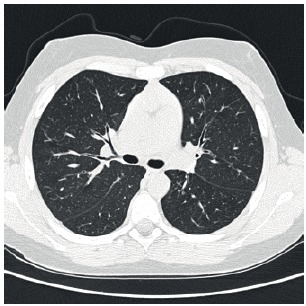
HRCT scan of the chest showing pronounced ground-glass changes in both lung fields, consistent with extrinsic allergic alveolitis.

A 7-year-old boy, whose father and paternal grandmother were under investigation for suspected HP, presented with a personal history of *situs inversus totalis* and recurrent respiratory infection. The boy was admitted to the emergency room with a 5-month history of productive cough, accompanied by progressively worsening dyspnea on exertion, with no improvement with bronchodilators and inhaled corticosteroids. It was reported that there had been a parakeet at home since the onset of the symptoms, and that the home also had a henhouse and a cooperage. The initial examination revealed an SpO_2_ of 84% on room air, subcostal retraction, and bilateral crackles. Ancillary test results were as follows: ESR, 30 mm/h; PCR, 1.41 mg/dL; IgG, IgA, and IgM levels, increased; serologies for *Mycoplasma pneumoniae, Legionella pneumophila*, and* Chlamydia pneumoniae* (IgG and IgM), negative; chest X-ray findings, bilateral diffuse perihilar interstitial infiltrate; sweat test, 32 mEq/L; PPD, 0 mm induration; serum precipitins to birds droppings and feathers, negative; echocardiogram, *situs inversus totalis*; chest HRCT findings, pronounced ground-glass changes in both lung fields, consistent with extrinsic allergic alveolitis (Figure 3); PFT, indicative of severe obstructive lung disease that was unresponsive to bronchodilators; BAL fluid findings, an intense lymphocytic alveolitis and neutrophilia, with a marked predominance of CD8+ (CD4/CD8 ratio < 0.1%). The patient received inhaled corticosteroids for 2 years, which resulted in progressive clinical and radiological improvement.

## Discussion

The patients described had a family history of HP accompanied by a clinical profile that corresponded to the subacute form of HP, with an obstructive pattern and symptoms such as cough, dyspnea on exertion, weight loss, and sometimes fever, the onset of which occurred within weeks to months of exposure to an antigen.

A high index of suspicion was essential for the diagnosis, as were analytical and laboratory criteria. Among the major criteria, defined by Schuyler et al.,^(^ ^7^ ^)^ are symptoms consistent with the disease, evidence of exposure to antigens as per clinical history, X-ray and CT findings consistent with HP, and lymphocytosis in BAL fluid. Chief among the minor criteria are crackles at the lung bases and arterial hypoxemia. In only one of the patients was there a decrease in DLCO. In none of the cases was it possible to detect precipitating antibodies to the antigen. Although they are objective markers of exposure, negative results do not absolutely exclude the diagnosis.^(^ ^1^ ^-^ ^4^ ^,^ ^6^ ^)^

In these three cases, it was possible to detect a diffuse reticulonodular/interstitial pattern on chest X-ray, as well as a ground-glass pattern, ground-glass areas, and micronodules, characteristic of the subacute form.

The detection of lymphocytosis in the BAL fluid from the patients, as well as the finding of a CD4/CD8 ratio < 0.1% in two of them, lent support to the diagnosis of HP.

Given that the disease was not severe in any of the cases, the treatment consisted in avoiding contact with the allergen and using oral/inhaled corticosteroids, which resulted in progressive clinical improvement, as well as in improvement in radiological and ventilatory parameters.

The need for lung biopsy should be weighed in terms of its cost-benefit ratio, and it should be considered in rare cases in which there is diagnostic uncertainty or in which the clinical course or treatment response is unclear.^(^ ^3^ ^)^

HP is an uncommon disease in children and has nonspecific symptoms. For the diagnosis of HP, it is important that clinical evaluation be performed and allergen exposure be investigated. In all three cases described above, the patients had a family history of HP, although they were not screened for the presence of HLA. These facts lead us to believe that, although allergens are the major triggering factor for this disease, heredity is also an important cofactor. Early diagnosis of HP is crucial in order to prevent severe and irreversible complications, such as pulmonary fibrosis.
