# Supplementary material for: The value of family history in the diagnosis of hypersensitivity pneumonitis in children
Source: J Bras Pneumol. 2014 Mar-Apr;40(2):183–7. doi: 10.1590/S1806-37132014000200013 (PMC4083639; doi:10.1590/S1806-37132014000200013)
Supplement: Translation

# Translation: O valor da história familiar no diagnóstico de pneumonite de hipersensibilidade em crianças*

**Authors:** Joana Cardoso, Isabel Carvalho

**Affiliations:** Centro Hospitalar de Vila Nova de Gaia/Espinho, Porto, Portugal; Centro Hospitalar de Vila Nova de Gaia/Espinho, Porto, Portugal

**Keywords:** Alveolite alérgica extrínseca, Lavagem broncoalveolar, Glucocorticoides

## Abstract

A pneumonite de hipersensibilidade (PH), ou alveolite alérgica extrínseca, é uma doença imunologicamente mediada, resultante da inalação de substâncias orgânicas que desencadeiam uma reação inflamatória na parede dos alvéolos, bronquíolos e interstício em indivíduos susceptíveis. Apesar de ser uma doença ocupacional de predomínio na idade adulta, estão descritos casos em crianças. O diagnóstico de PH requer grande suspeição, e seu tratamento consiste na ausência de contato com o antígeno e, em alguns casos, pode ser necessária corticoterapia sistêmica, evitando-se a progressão para fibrose pulmonar. Relatamos três casos clínicos de crianças com história de contato com aves e história familiar de PH. Todos os casos se apresentaram com tosse e dispneia aos esforços. O diagnóstico foi possível por história clínica e exames auxiliares de diagnóstico compatíveis, incluindo lavado broncoalveolar com predomínio de linfócitos (> 60%, especialmente linfócitos T CD8+) e TCAR de tórax com padrão em vidro fosco. O diagnóstico precoce é fundamental na PH para se prevenir a evolução para fibrose pulmonar. Fatores hereditários parecem influenciar seu aparecimento.

## Introdução

A pneumonite de hipersensibilidade (PH) ou alveolite alérgica extrínseca é uma doença mediada imunologicamente e causada pela inalação de substâncias orgânicas que, em indivíduos susceptíveis, desencadeia uma reação inflamatória na parede de alvéolos, bronquíolos e interstício. ^(^ ^1^ ^-^ ^8^ ^)^ Apesar de ser uma doença ocupacional de predomínio na idade adulta, estão descritos casos em crianças, na sua maioria após a exposição a proteínas aviárias.^(^ ^4^ ^,^ ^6^ ^)^

A prevalência varia de 5-15% nos indivíduos expostos a um alérgeno,^(^ ^1^ ^-^ ^3^ ^)^ e a frequência da doença relaciona-se com vários fatores (quantidade de alérgeno inalado, duração da exposição, natureza do antígeno e resposta imune do hospedeiro).^(^ ^1^ ^,^ ^2^ ^)^ A hereditariedade pode ser importante, se verificando maior predisposição em famílias com HLA-DR7, HLA-B8 e HLA-DQw3 positivos.^(^ ^2^ ^,^ ^6^ ^)^

A PH pode ser classificada segundo a sintomatologia (aguda, subaguda ou crônica) ou segundo a natureza dinâmica da doença (aguda progressiva, aguda intermitente não progressiva ou doença recorrente não aguda).^(^ ^1^ ^-^ ^3^ ^,^ ^5^ ^-^ ^6^ ^,^ ^8^ ^)^ A forma aguda corresponde a uma exposição intermitente e intensa ao alérgeno, que se assemelha a uma infecção respiratória de etiologia viral que resolve ao fim de 24-48 h.^(^ ^1^ ^-^ ^3^ ^,^ ^5^ ^-^ ^7^ ^)^ A forma subaguda se caracteriza por dispneia aos esforços, astenia e perda ponderal, correspondendo a uma exposição continuada mas menos intensa ao alérgeno.^(^ ^1^ ^-^ ^3^ ^,^ ^5^ ^-^ ^6^ ^)^ A forma crônica se caracteriza pela progressão para lesão pulmonar irreversível, com fibrose pulmonar,^(^ ^1^ ^,^ ^3^ ^)^ correspondendo à inalação insidiosa e prolongada de baixas concentrações de antígeno, sem quadro agudo prévio.^(^ ^1^ ^-^ ^3^ ^,^ ^5^ ^-^ ^7^ ^)^

Não existe nenhum exame auxiliar de diagnóstico patognomônico, sendo a hipótese diagnóstica colocada perante um elevado índice de suspeição, história clínica, exame objetivo, estudo laboratorial, provas de função respiratória (PFR) e estudo de imagem.^(^ ^1^ ^-^ ^4^ ^,^ ^6^ ^)^

Os dados laboratoriais são inespecíficos - leucocitose, aumento da VHS e aumento dos níveis de proteína C reativa (PCR) e de imunoglobulinas. ^(^ ^1^ ^,^ ^2^ ^,^ ^4^ ^)^ Os testes cutâneos e a presença de anticorpos precipitantes para o antígeno são marcadores de exposição, mas a sua negatividade não exclui o diagnóstico.^(^ ^1^ ^-^ ^4^ ^,^ ^6^ ^)^ As provas de provocação mediante a inalação do antígeno predisponente são o melhor método diagnóstico na persistência de dúvidas diagnósticas, mas essas devem ser realizadas em meio hospitalar.^(^ ^1^ ^-^ ^4^ ^,^ ^6^ ^)^

As PFR mostram geralmente um padrão restritivo, caracterizado por uma diminuição da CVF e da CPT. Na fase crônica é possível encontrar um padrão obstrutivo, estando a DLCO geralmente diminuída.^(^ ^1^ ^-^ ^4^ ^,^ ^6^ ^)^

O lavado broncoalveolar (LBA) apresenta um predomínio de linfócitos (> 60%), nomeadamente os linfócitos T supressores (CD8+), com uma diminuição do índice CD4/CD8 < 1% (raramente encontrado em crianças).^(^ ^1^ ^-^ ^4^ ^,^ ^6^ ^)^

As alterações na radiografia pulmonar surgem em função do grau de doença e relacionam-se pouco com a gravidade dos sintomas, variando de normal a um padrão nodular/reticulonodular (na forma aguda e subaguda).^(^ ^1^ ^-^ ^6^ ^)^ A TCAR de tórax pode mostrar um padrão micronodular difuso (fase aguda) ou enfisema obstrutivo, lesões fibróticas intersticiais e imagens em vidro fosco (fase crônica),^(^ ^1^ ^-^ ^7^ ^)^ sendo o exame de maior utilidade no diagnóstico.

O tratamento consiste na ausência de contato com o antígeno, podendo ser o único tratamento nas formas agudas. A corticoterapia sistêmica é o tratamento de escolha nas formas subagudas e crônicas.^(^ ^1^ ^-^ ^4^ ^,^ ^6^ ^)^ O prognóstico varia da recuperação completa nas formas agudas e não progressivas à fibrose pulmonar nas formas crônicas. O grau de fibrose pulmonar no momento do diagnóstico é o principal fator prognóstico.^(^ ^1^ ^,^ ^2^ ^)^

## Relato de casos

Caso 1

Relatamos o caso de um menino de 12 anos com história de asma e história familiar de PH. Residente em zona rural, tinha contato com pombos e canários, que agravavam os sintomas. Chegou ao serviço de urgência com tosse produtiva e febre com 2 dias de evolução, associadas a anorexia e perda ponderal. A radiografia torácica revelou um infiltrado peri-hilar bilateral, e o paciente teve alta após ser medicado com claritromicina, que foi substituída pela associação amoxicilina e ácido clavulânico por persistência da sintomatologia 5 dias mais tarde. Um mês depois, por manter tosse intermitente com períodos de agravamento e dispneia aos pequenos esforços, o paciente foi novamente atendido, apresentando cianose peribucal, palidez cutânea, hipoxemia (SpO_2_ = 85% em ar ambiente), tiragem global e diminuição global dos sons respiratórios, com estertores nas bases. Os exames complementares revelaram VHS de 36 mm/h; PCR de 22,8 mg/L; níveis de IgA e IgG elevados; teste Phadiatop^(r)^ (Phadia, Uppsala, Suécia) inalatório e alimentar negativo; PPD de 0 mm; teste do suor de 34 mEq/L; radiografia torácica com infiltrado peri-hilar e basal de padrão reticulonodular bilateralmente (Figura 1); ecocardiograma sem alterações; TCAR de tórax com consolidação do espaço aéreo e broncograma aéreo em ambos os lobos inferiores, associados a padrão intersticial bilateral de distribuição difusa, padrão centrilobular com alguns micronódulos e áreas em vidro fosco; sorologia para *Chlamydophila psittaci* negativa; PFR indicando alteração ventilatória obstrutiva leve; defeito moderado na DLCO; e LBA com alveolite linfocítica intensa, com franco predomínio de CD8+ (relação CD4/CD8 < 0,3%). Realizou corticoterapia oral durante um ano com resolução completa da sintomatologia e das lesões à TCAR de tórax, bem como melhoria nos resultados de PFR ao fim de três anos de seguimento.

**Figura 1:** Radiografia de tórax revelando infiltrado peri-hilar e basal de padrão reticulonodular bilateralmente.

## Caso 2 

Uma menina de 14 anos, residente em zona rural, com pássaros no domicílio e história familiar de PH. A paciente estava saudável até novembro de 2002, quando recorreu ao serviço de urgência por febre e tosse; ao exame objetivo, apresentava estertores e dificuldade respiratória. Realizou radiografia torácica, que revelou infiltrado intersticial. A paciente recebeu alta, sendo medicada com claritromicina e prednisolona. Retornou ao serviço ambulatorial uma semana depois, referindo dispneia aos pequenos esforços. Os exames complementares revelaram VHS de 24 mm/h; aumento do nível de IgG, mas sem alterações das outras imunoglobulinas; sorologias para Mycoplasma pneumoniae e Chlamydia pneumoniae negativas; IgG e IgM negativas; radiografia torácica com aumento da trama broncovascular bilateralmente e infiltrado intersticial difuso de predomínio peri-hilar bilateral; TCAR de tórax com tênue adensamento do parênquima pulmonar de ambos os pulmões com distribuição ligeiramente heterogênea, revelando pequenos nódulos de limites mal definidos e alterações em vidro fosco (Figura 2); ecocardiograma e eletrocardiograma sem alterações; LBA com alveolite linfocítica intensa e neutrofílica discreta, com franco predomínio de CD8+ (relação CD4/CD8 < 0,1%). A paciente foi mantida sob corticoterapia inalatória durante 2 anos, com melhoria clínica progressiva.

**Figura 2:** TCAR de tórax revelando tênue adensamento do parênquima pulmonar de ambos os pulmões, com distribuição heterogênea; pequenos nódulos de limites mal definidos e alterações em vidro fosco.

## Caso 3

Um menino de 7 anos, cujos pai e avó paterna estavam sendo investigados pelo setor de pneumologia por suspeita de PH. Tinha história pessoal de *situs inversus totalis* e infecções respiratórias de repetição. Deu entrada no serviço de urgência por tosse produtiva com cinco meses de evolução, associada a dispneia aos esforços e de agravamento progressivo, sem melhoria com broncodilatador e corticoterapia inalatória. Foi relatada a existência de um periquito no domicílio desde o início da sintomatologia, bem como um galinheiro e uma oficina de tanoaria. Ao exame inicial, apresentava SpO_2_ de 84% em ar ambiente, tiragem subcostal e estertores bilaterais. Os exames complementares revelaram VHS de 30 mm/h; PCR de 1,41 mg/dL; aumento dos níveis de IgG, IgA e IgM; sorologias para Mycoplasma pneumoniae, Legionella pneumophila e Chlamydia pneumoniae (IgG e IgM) negativas; radiografia torácica com infiltrado peri-hilar e intersticial difuso bilateral; teste do suor de 32 mEq/L; PPD de 0 mm; precipitinas séricas para fezes e penas de aves negativas; ecocardiograma com situs inversus completo; TCAR de tórax com exuberantes alterações em vidro fosco em ambos os campos pulmonares, compatível com alveolite alérgica extrínseca (Figura 3); PFR indicando alteração obstrutiva grave sem resposta ao broncodilatador; LBA com alveolite linfocítica intensa e neutrofilia, com franco predomínio de CD8+ (relação CD4/CD8 < 0,1%). O paciente recebeu corticoterapia inalatória durante 2 anos, com melhoria clínica e radiológica progressiva.

**Figura 3:** TCAR de tórax revelando exuberantes alterações em vidro fosco em ambos os campos pulmonares, compatível com alveolite alérgica extrínseca.

## Discussão

Os pacientes descritos apresentavam história familiar de PH associada a um quadro clínico correspondente à forma de apresentação subaguda de PH, com padrão obstrutivo e sintomas como tosse, dispneia aos esforços, perda ponderal e, por vezes, febre, sintomas esses desencadeados ao fim de algumas semanas a meses de exposição a um antígeno.

Para o diagnóstico foi essencial um elevado índice de suspeição, bem como critérios analíticos e laboratoriais. Entre os critérios maiores, definidos por Schuyler et al.,^(^ ^7^ ^)^ encontram-se os sintomas compatíveis, a evidência de exposição a antígeno mediante a história clínica, os achados radiológicos na radiografia e na TC compatíveis com PH e a presença de linfocitose no LBA. Dentre os critérios menores, destacam-se a existência de estertores nas bases e hipoxemia arterial. Apenas em um dos pacientes se documentou uma diminuição da DLCO. Em nenhum dos casos foi possível identificar anticorpos precipitantes para o antígeno. Apesar de esses serem marcadores objetivos de exposição, a sua negatividade não exclui em absoluto o diagnóstico.^(^ ^1^ ^-^ ^4^ ^,^ ^6^ ^)^

Nesses três casos foi possível verificar a presença de um padrão reticulonodular/intersticial difuso na radiografia torácica, bem como a existência de um padrão em vidro fosco, áreas em vidro fosco e micronódulos, característicos da forma subaguda.

A presença de linfocitose no LBA dos pacientes, bem como a existência de uma relação CD4/CD8 < 0,1% em dois deles, permitiu corroborar o diagnóstico de PH.

Dada a inexistência de forma grave da doença, o tratamento instituído em todos os pacientes foi a ausência de contato com o alérgeno e a utilização de corticoterapia oral/inalatória, que resultou em melhoria clínica progressiva, assim como a dos parâmetros ventilatórios e radiológicos.

A realização de biópsia pulmonar deve ser ponderada mediante sua relação custo-benefício, devendo ser considerada em raros casos nos quais haja dúvida no diagnóstico ou naqueles cuja evolução clínica ou resposta ao tratamento seja dúbia.^(^ ^3^ ^)^

A PH é uma patologia pouco frequente em crianças e apresenta sintomas inespecíficos. Para o seu diagnóstico, é importante a clínica e a investigação de exposição a alérgenos. Em todos os casos descritos havia história familiar de PH, apesar de não ter sido investigada a presença de HLA. Esses fatos levam-nos a acreditar que apesar de o alérgeno ser o principal fator desencadeante dessa patologia, a hereditariedade também se apresenta como um importante cofator. É fundamental o diagnóstico precoce de PH, o que permite se evitar complicações irreversíveis e graves, como a fibrose pulmonar.
